# Odontogenic (hematogenic) or sinusopathy (contiguous) brain abscess: Case report

**DOI:** 10.4317/jced.61707

**Published:** 2024-07-01

**Authors:** Claudine Thereza-Bussolaro, Eduarda B. Ramos, Anna LS. Yanai, Luiz-Evaristo-Ricci Volpato, Alexandre M. Borba

**Affiliations:** 1 School of Dentistry, University of Cuiabá, Cuiabá, MT, Brazil; 2 School of Dentistry, Anhanguera University, Sinop, MT, Brazil; 3 School of Medicine, Federal University of Mato Grosso, Sinop, MT, Brazil; 4 Department of Dentistry, Mato Grosso Cancer Hospital, Cuiabá, MT, Brazil

## Abstract

Brain abscess is a rare infectious condition, affecting 0.4 to 0.9 per 100,000 individuals annually, with classic symptoms of fever, headache, and neurological deficits. The origin can be contiguous, hematogenous, due to ruptures of brain barriers, or cryptogenic. Dental infections, such as those related to Gemella morbillorum, are atypical, and when related to odontogenic sinusitis, it is normally unilateral. This report describes a case of peculiar brain abscess, of unconfirmed source, possibly involving sinusitis or periapical odontogenic lesion in an immunocompetent young woman. A 22-year-old patient presented with sinusitis showed by computed tomography, progressing to a brain abscess caused by multidrug-resistant Streptococcus sanguis. Additional cultures revealed Gemella morbillorum in maxillary sinusitis. Treatment involved stereotactic drainage, sinusotomy, and prolonged antibiotic therapy, with recurrence and surgical reintervention, in addition to prophylactic dental extraction and exerese of the brain cyst capsule. Brain abscess represents a significant medical challenge, often posing difficulties in pinpointing its primary infectious source despite the aid of comprehensive laboratory and imaging diagnostics, as evidenced in this case. Timely and targeted intervention in preceding infections assumes paramount importance for effective management, underscoring the indispensable role of a multidisciplinary healthcare team. Active patient engagement and adherence to treatment protocols are imperative to mitigate complications and foster favorable disease progression.

** Key words:**Brain Abscess, Dental Focal Infection, Gemella, Sinusitis, Streptococcus sanguis.

## Introduction

Brain abscess is an uncommon infectious process, affecting 0.4 to 0.9 per 100,000 individuals per year (1). It is defined as a localized collection, and its evolution depends on the patient’s immune response as well as the potential of the infecting pathogen. Fever, headache, and neurological deficits characterize its classic triad (2). Such abscess can originate from contiguous dissemination, hematogenous dissemination, ruptures of brain barriers, or cryptogenic. Cryptogenic, is referred when the source of infection cannot be defined (2), corresponding to 25% of cases (3).

Paranasal sinusitis and dental infections are responsible, respectively, for 10% and 2% of cases of contiguous dissemination, mainly affecting the frontal lobes. The usual pathogens for both primary infections are *Streptococci*, *Staphylococci*, and *Bacteroides* spp (4). Accordingly to the literature (5), brain abscesses caused by *Gemella morbillorum*, 4 (four) out of 9(nine) patients have a history of dental infections or dental procedures, 2(two) did not have the primary source described, one had been originated from septic arthritis, another from pleuritis, and the last due to maxillary sinusitis (5).

It is crucial to underscore that maxillary sinusitis can arise as a secondary consequence of dental infections, irrespective of its association with the development of brain abscesses, a condition called odontogenic sinusitis (OS). Characteristically unilateral, OS accounts for a noTable proportion, ranging from 45% to 75%, of unilateral maxillary sinus opacifications observed on computed tomography (CT) scans (3).

The primary objective of this study is to present a clinical case involving concurrent manifestations of brain abscess, pansinusitis, and periapical odontogenic infection, thereby emphasizing the critical importance of comprehensive evaluation of the entire cranioencephalic complex for facilitating optimal management and minimizing the risk of complications.

## Case Report

A 22-year-old young female was attended in the emergency room of a private hospital in Sinop, MT, Brazil, with complaints of facial pain. CT scans of the face revealed pansinusitis, and a periapical lesion at the upper left second molar (tooth #27). She received an oral prescription of Amoxicillin 875 milligrams (mg) plus Potassium Clavulanate 125 (mg) every 12 hours and was discharged from the hospital.

**Figure 1:**
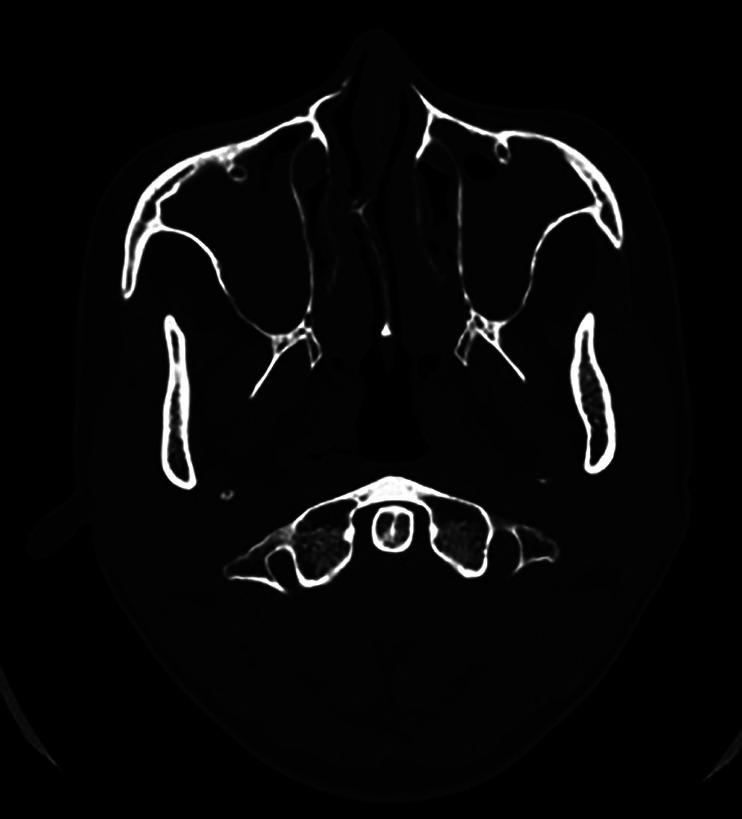
CT scan after 15 days showing the persistence of bilateral sinusopathy in an axial view.

Fifteen days later (D 15), the patient attended a follow-up appointment, and a new CT scan was performed where the bilateral sinus disease persisted (Fig. 1). The patient returned home with oral therapeutic prescription, but on D 31 she returned to the hospital complaining of headache and was then admitted.

**Figure 2:**
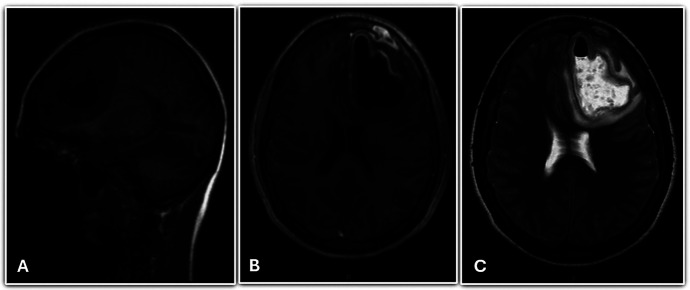
MRI showing A) abscess in the left frontal region; B) expansive formation in the left frontal lobe and frontal sinus discontinuity; and C) hypersignal in the adjacent parenchyma and shift of the midline to the right (contrast).

After three days (D 34), the patient presented a fluctuation in consciousness and periods of intense drowsiness. Magnetic resonance imaging (MRI) of the skull confirmed the presence of an abscess in the left frontal region, expansive formation in the left frontal lobe, frontal sinus discontinuity, hypersignal in the adjacent parenchyma, and midline shift to the right (Fig. 2). The patient underwent urgent stereotactic drainage (D 34), the culture of which showed multidrug-resistant *Streptococcus sanguis*, sensitive to vancomycin. An urgent sinusotomy was also performed, in which a culture of the material collected from the maxillary sinus grown *Gemella morbillorum*. Patient was sent to the intensive care unit (ICU) where intravenous antibiotic was maintained postoperatively.

**Figure 3:**
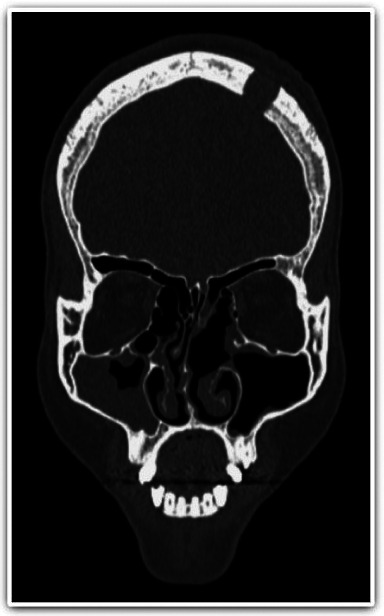
Coronal CT showing resolution of sinusopathy on the operated left side and persistence of sinusopathy on the right side.

The facial CT performed 14 days after the diagnosis of the brain abscess (D 48) indicated remission of the left maxillary sinusitis, but maintenance of the right maxillary sinusitis (Fig. 3).

**Figure 4:**
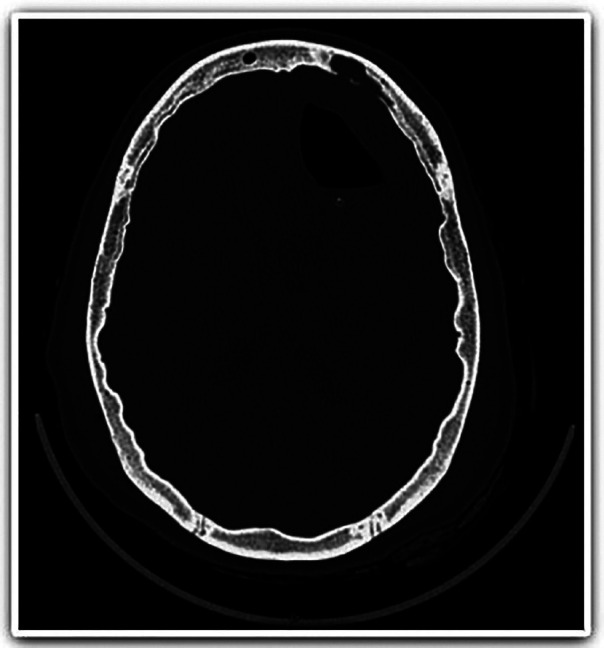
MRI revealing hyposignal compatible with brain abscess.

The patient was being covered by health insurance that suggested she should be transferred to home care, as she was admitted to the hospital only for intravenous antibiotic therapy. However, the patient did not cooperate with the home-care treatment, resulting in readmission due to vomiting and headache after four days at home. A new MRI was performed (Fig. 4) showing recurrence of the brain abscess, requiring surgical reintervention. In this second surgical procedure, the brain abscess was drained, and in the following day, teeth #18, 27, and 28 were extracted concomitant to bilateral sinusotomy plus material collection for culture. The culture result was negative, and the patient progressed well during hospitalization.

Thirty-four days (D 68) after the first drainage, the patient underwent surgery to remove the cerebral cyst capsule. The patient was discharged from the hospital only after completing antimicrobial treatment, without presenting any neurological deficit. The sequence of procedures and medication management can be seen in  Table 1. The patient agreed to the description and publication of her clinical case by signing an Informed Consent Form.

## Discussion

We present a case regarding a brain abscess of conflicting etiology in a young immunocompetent patient in which the etiology of the abscess cannot be defined. This focal pyogenic infection of the brain parenchyma has the presence of a contiguous focus of infection, trauma, and hematogenous dissemination from a distant focus as it main predisposing and associated factors. Microbial etiology depends on the site of primary infection, patient’s age, underlying condition, and immunological status (1). The most common brain abscesses generally affect middle-aged, male patients with comorbidities, especially vascular (6). However, we present an atypical case of a young, female patient with no comorbidities.

The etiology of the abscess in the case presented is conflicting due to the development of the condition after an episode of pansinusitis. Initially, the possibility of OS was considered due to a hypodense image at the root apex of tooth 27. However, the patient had no odontogenic infection or clinical signs compatible with OS. The discrete periapical lesion suggestive of periapical granuloma in root tooth 27 was not in line with the diagnosis of OS, as there were no signs of infection or cortical disruption of the left maxillary sinus. Furthermore, the patient did not present the typical symptoms associated with OS, such as an unpleasant mouth odor or toothache. Complaints from the beginning of hospitalization were headache and sinusitis, without evidence of purulent content during the second sinusotomy surgery, contradicting what was expected in the case of OS (3).

The patient’s initial CT scan showed pansinusitis, a new CT scan fifteen days later revealed the persistence of pansinusitis even after ATB therapy. It showed bilateral opacifications in the sphenoid, ethmoid, frontal, and maxillary sinuses, suggestive of a case more aligned with chronic rhinosinusitis (3). Nine days after this second CT, the MRI and CT images revealed the brain abscess and maxillary sinusitis exclusively on the left side. Regarding microbiology, the microorganisms most isolated in sinusitis are anaerobic bacteria, aerobic and microaerophilic *streptococci*, *Enterobacteriaceae* and *Staphylococcus aureus* (1,2,6). In the case presented here, *Streptococcus sanguis*, currently known as *Streptococcus sanguinis*, was in the brain abscess and *Gemella* was in the sinus secretion. *Streptococcus sanguinis* is a microorganism particularly located in dental plaque that lodges in the heart valve, leading to bacterial endocarditis, commonly related to patients’ poor dental hygiene (7). Brain abscesses caused by *Streptococcus sanguinis* are rare, only seven cases were reported in the literature (8). In the case presented, the patient denied having undergone dental procedures, but had an odontogenic infection that could have spread through the hematogenous route to the brain tissue. Furthermore, the bacteria found in the maxillary sinus differed from that found in the brain abscess. Rhinosinusitis can be infected by microorganisms from its microbiota, corroborating the case presented, since *Gemelli’s* natural habitat is the oral cavity and upper airways (3,5).

Treatment of brain abscesses includes clinical and surgical approaches. Abscesses larger than 2 cm are generally treated surgically, and associated with prolonged antimicrobial treatment (1,9). The case presented here was larger than 2 cm and was treated surgically and pharmacologically. Before encapsulation and localization of the abscess, antimicrobial therapy, accompanied by measures to control the increase in intracranial pressure, is essential, as was done in the reported case. After an abscess forms, surgical excision or drainage combined with prolonged antibiotics (usually 4-8 weeks) remains the treatment of choice (1,2). Specimens obtained during surgery or aspiration guided by stereotactic CT should be sent for aerobic, anaerobic, mycobacterial, and fungal culture as well as, when indicated, for protozoa (1,5,9).

Although it is not always recommended, in this case, it was decided to extract all condemned teeth as a way of preventing infections given the patient’s serious condition. The empirical antibiotic regimen for brain abscesses includes the use of metronidazole, cephalosporin, and vancomycin (1,6). The case presented was initially treated with vancomycin, metronidazole and carbapenem and after confirmation of susceptibility by culture and antibiogram examination, the prescription was maintained. Weekly imaging control via CT or MRI was followed to assess the evolution of the case and response to therapy, following current recommendations (3,9,10).

Most cases of brain abscess resolve after initiation of empiric antibiotic therapy (2,6). The prognosis usually worsens when the capsule ruptures, resulting in ventriculitis and meningism (2). Therefore, effective management by removing the capsule, as in the case presented here, is essential for a good prognosis.

## Conclusions

Brain abscess is a serious complication and, sometimes, it can be difficult to confirm its primary infectious origin, even with the support of laboratory and imaging tests. The reported case initially presented itself as pansinusitis progressing to brain abscess with oral microbiota bacteria, treated with a combination of surgical and pharmacological treatment.

## Figures and Tables

**Table 1:** Sequence of treatment performed on the patient.

DAY	TREATMENT/PROCEDURE
D1	Ambulatory consultation (ER) + CT with images of pansinusitis + oral ABT
D 15	Follow up_CT - pansinusitis + medication Amoxicillin + Clavulanate
D 31	Hospitalization- Medication
	-Vancomycin 1g (IV) every 12 hours
	-Metronidazole 500mg (IV), every 8 hours
	-Meropenem 2g (IV)
	-Dexamethasone 4mg (IV) every 12 hours
D 34	New CT - maxillary sinusitis L
	MRI- diagnosis of brain abscess
	Stereotactic drainage + sinusotomy
D 48	Oral evaluation​
D 52	New MRI
D 53	Hospital discharge and transfer to home care
D 57	New MRI
D 59	Stereotactic drainage
D 60	Bilateral sinusotomy and dental extractions
D 68	Treatment by surgical excision of the cerebral cyst capsule
D 92	Discharge after treatment completion

D, day; ER, emergence room; CT, computed tomography; ABT, antibiotic therapy; IV, intravenous; mg, milligrams; gr, grams; L, left; MRI, magnetic resonance imaging

## Data Availability

The datasets used and analyzed in the current study are available from the corresponding author upon reasonable request.
